# Assessing quality of *Medicago sativa* silage by monitoring bacterial composition with single molecule, real-time sequencing technology and various physiological parameters

**DOI:** 10.1038/srep28358

**Published:** 2016-06-24

**Authors:** Weichen Bao, Zhihui Mi, Haiyan Xu, Yi Zheng, Lai Yu Kwok, Heping Zhang, Wenyi Zhang

**Affiliations:** 1 Key Laboratory of Dairy Biotechnology and Engineering, Ministry of Education, Inner Mongolia Agricultural University, Hohhot, Inner Mongolia 010018, China

## Abstract

The present study applied the PacBio single molecule, real-time sequencing technology (SMRT) in evaluating the quality of silage production. Specifically, we produced four types of *Medicago sativa* silages by using four different lactic acid bacteria-based additives (AD-I, AD-II, AD-III and AD-IV). We monitored the changes in pH, organic acids (including butyric acid, the ratio of acetic acid/lactic acid, γ-aminobutyric acid, 4-hyroxy benzoic acid and phenyl lactic acid), mycotoxins, and bacterial microbiota during silage fermentation. Our results showed that the use of the additives was beneficial to the silage fermentation by enhancing a general pH and mycotoxin reduction, while increasing the organic acids content. By SMRT analysis of the microbial composition in eight silage samples, we found that the bacterial species number and relative abundances shifted apparently after fermentation. Such changes were specific to the LAB species in the additives. Particularly, *Bacillus megaterium* was the initial dominant species in the raw materials; and after the fermentation process, *Pediococcus acidilactici* and *Lactobacillus plantarum* became the most prevalent species, both of which were intrinsically present in the LAB additives. Our data have demonstrated that the SMRT sequencing platform is applicable in assessing the quality of silage.

*Medicago sativa* is one of the most important forage crops that are widely used in feeding dairy cows^1^,^2^. It contains essential nutrient ingredients for cattle, including proteins, vitamins and minerals^3^. In China, most planting areas of *M. sativa* are located in the monsoon regions. The frequent rains in these regions do not only reduce the nutritional value of the crops, but also increase the risk of proliferation of undesirable microbes. To make the best use of *M. sativa*, there is a growing interest in improving the ensilage technology. Most published researches address the agronomical, biochemical, microbiological, nutritional and engineering aspects of the process^4^. Among them, recent successes have been achieved by incorporating various additives to aid the fermentation process, which has become a common practice^5^.

The assessment of silage quality is typically based on determining the fermentation qualities and changes in microbial compositions^6^. During the past few years, the evaluation of silage quality relied on a comprehensive range of chemical analysis^7^. Common silage quality indicators include the silage dry matter weight and content, water-soluble carbohydrate concentration, and target bacterial counts^8^. Although these indicators may partially reflect the silage quality, the assessment process is time-consuming and the results are sometimes inaccurate, especially because of the limited information obtained regarding the silage microbial composition. Recently, real-time polymerase chain reaction and in-depth 16S rRNA gene libraries sequence analysis have been designed to quantify the bacterial microbiota of silages prepared with or without commercial inoculants^9^,^10^. These studies have demonstrated the applicability of using such molecular techniques in quantifying certain silage-specific species under a wide variety of conditions. However, the silage bacterial microbiota profiles described by the previous studies are restricted to the genus precision due to the relatively low taxonomical resolution of the traditional DNA sequencing technique that could only determine the partial sequence of the 16S rRNA gene.

The Pacific Biosciences (PacBio) single molecule, real-time sequencing technology (SMRT) is able to depict the bacterial profile of target samples to species level precision because of its power in generating long sequence reads^11^,^12^. In the present study, apart from analyzing the silage quality using conventional indicators like pH, organic acid contents, and mycotoxin formation, we specifically focused on detecting and comparing the bacterial microbiota composition of *M. sativa* silages produced by adding four different lactic acid bacteria (LAB) additives using the PacBio SMRT method. Our data have demonstrated that the SMRT sequencing platform is suitable for assessing the quality of silage.

## Results

### Fermentative changes in silage pH and organic acids content

**Figure 1:**
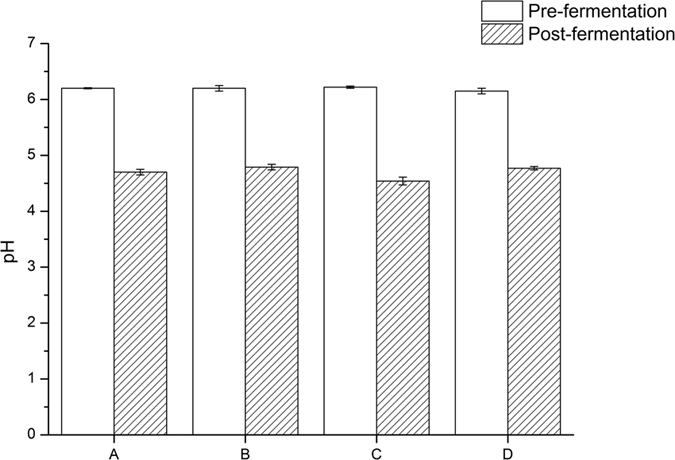
The changes in silage pH before and after fermentation.

The silage fermentative changes in pH and various organic acids including butyric acid, the ratio of acetic acid/lactic acid, γ-aminobutyric acid, 4-hyroxy benzoic acid and phenyl lactic acid are shown in Fig. 1 and Table 1. Generally, the addition of any of the four LAB additives (AD-I, AD-II, AD-III and AD-IV) resulted in varying degree of fermentative changes in pH and organic acids content. After fermentation, the silage inoculated with AD-IV had both elevated butyric acid concentration and acetic acid/lactic acid ratio. The highest concentration of γ-aminobutyric acid was found in the silage fermented with AD-I, while the lowest one was that fermented with AD-IV. The silage with AD-II had a relatively higher content of both 4-hyroxy benzoic acid and phenyl lactic acid.

### Changes in silage mycotoxin content after fermentation

The changes in the silage aflatoxin B1, vomitoxin and zearalonone concentrations are given in Table 2. The silage concentrations of the three investigated toxins generally decreased upon fermentation. However, some exceptions were found in the silages fermented with AD-III and AD-IV. The former had a slight increase in aflatoxin B1 concentration, while the latter had mild elevation in both aflatoxin B1 and zearalonone levels.

### Changes in microbial composition after silage fermentation

**Figure 2:**
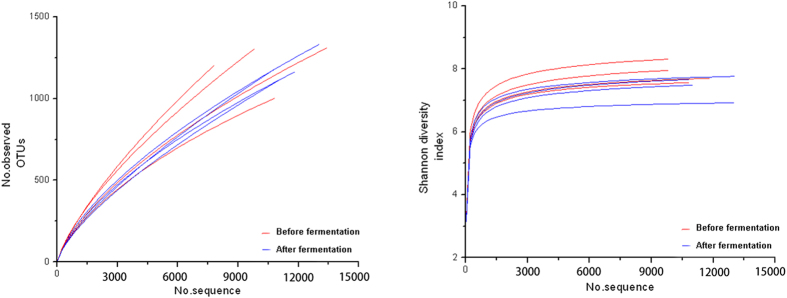
Shannon diversity index curves showing the diversity of taxa present in the dairy products.

SMRT sequencing of the full length 16S rRNA gene was performed to obtain accurate bacterial profiles of the silage samples at species level. A total of 67,199 raw reads were generated from 8 silage samples, with an average of 8,399 reads for each sample. The Shannon-Wiener diversity curves showed that the sequence depth was adequate for all samples (Fig. 2). Results from the Shannon index, Simpson index, Chao1 index and number of observed species (Table 3) indicated that most samples had a high bacterial biodiversity.

**Figure 3:**
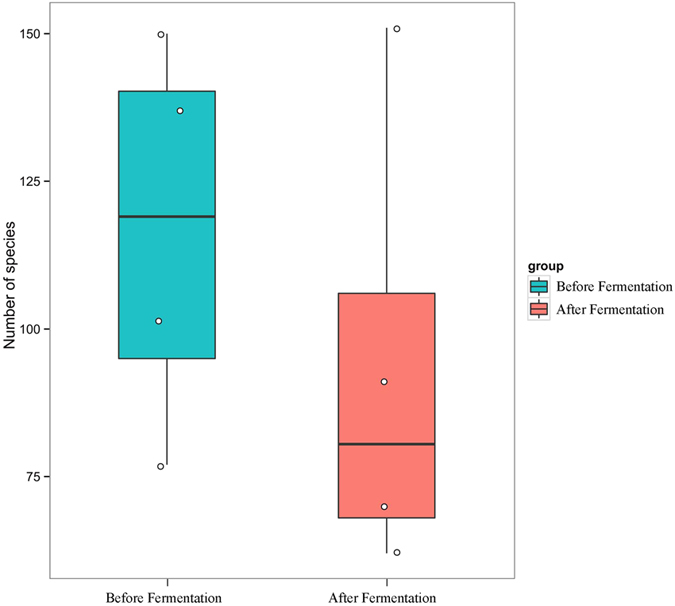
Box plots of the silage bacterial abundances at species level. Each dot represents a specific sample.

**Figure 4:**
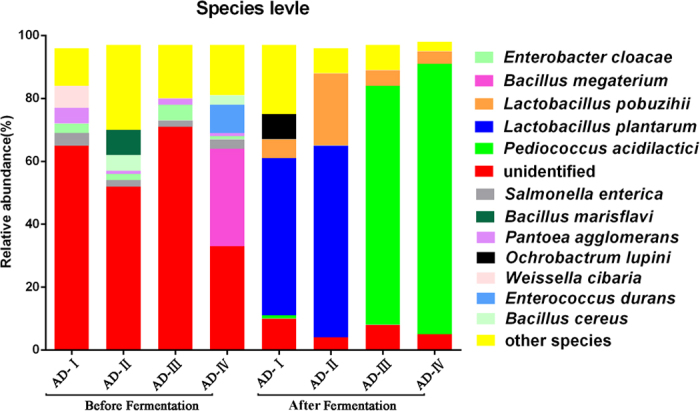
Relative abundances of the silage bacterial species before and after fermentation.

**Figure 5:**
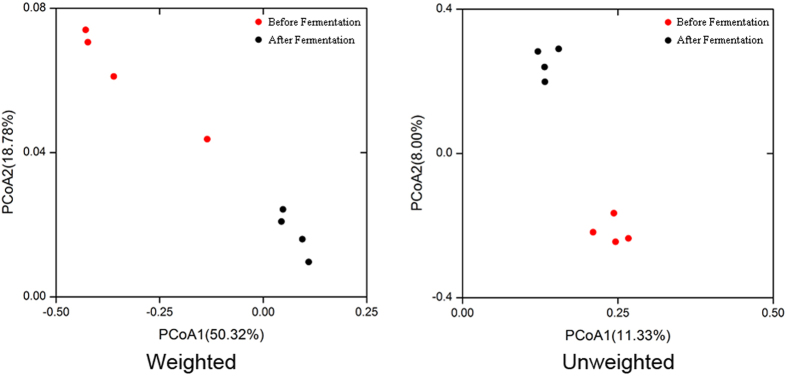
UniFrac weighted and unweighted principle coordinate analysis scores plot based on PC1 and PC2.

Using the RDP classifier, more than 960 species were identified from all samples; twelve of them had a relative abundance of >1%, namely *Pediococcus acidilactici*, *Lactobacillus plantarum*, *Lactobacillus pobuzihii*, *Bacillus megaterium*, *Salmonella enterica, Enterobacter cloacae*, *Pantoea agglomerans*, *Ochrobactrum lupini*, *Weissella cibaria*, *Enterococcus durans*, *Bacillus cereus* and *Bacillus marisflavi*. In fact, great variations were found among the species number (Fig. 3) and relative abundances of detected bacteria before and after fermentation (Fig. 4). Before fermentation, *Bacillus megaterium* was the most abundant species in the silage samples. After fermentation, *P. acidilactici* (41.18%) and *L. plantarum* (20.15%) appeared to be the dominant species in the silages (Fig. 4). Clearly, the prevalent species existed in the fermented silages were highly dependent on the original bacterial composition in the LAB additives. By PCoA analysis based on the weighted and unweighted UniFrac distances (Fig. 5), distinct clusters were identified in relation to the silage fermentation status.

## Discussion

Silages made from *M. sativa* are widely used in feeding dairy cows. To improve the fermentation, preservation and nutritional value of silages, LAB-containing additives are often added to aid the fermentative process. Good quality silages also maximize the feed efficiency and thus profitability. To ensure the quality of silages and its production process, it is therefore crucial to perform proper quality control. Traditionally, such evaluation is largely based on determining the microbial composition using biochemical methods, which is unsatisfactory, as the results may sometimes be difficult to interpret and ambiguous. In the present study, the full-length 16S rRNA gene-SMRT sequencing method was applied to monitor the quality of silage production.

Typically, the fermentation of *M. sativa* silage with LAB additives results in a decrease in pH due to the production of organic acids during the process. Commonly the drop in pH values was mainly caused by lactic acid production during fermentation; and a low pH is favorable, as the silages are better preserved and are more stable. In particular, acetic acid and butyric acid were found to increase the stability of the silage under aerobic conditions^13^. On the other hand, some compounds, e.g. butyric acid, were implicated as being responsible for reducing silage intake in a study published in 1963^14^. However, only moderate correlation was found between these fermentation acids and silage intake from a large number of animal trials^15^. Thus, it is still not entirely clear whether a high acidic content in the silage would affect the feed palatability; and whether the high concentration of butyric acid in the AD-IV-treated silage would have any negative effect on feeding remains to be further determined.

In addition to the aforementioned organic acids, 4-hyroxy benzoic acid and phenyl lactic acid have been shown to play a role in maintaining the quality of the silage by inhibiting the production of fungal mycotoxins^16^. Mycotoxins can cause nervous system disorders, vomiting, loss of appetite, immune function reduction, abortion, stillbirth and monster in cows^17^,^18^. Moreover, they can diminish dairy cattle feed intake and milk yield, and even lead to death. There is also an increased health risk for human after consuming milk derived from cows that have ingested contaminated feeds^19^. It is therefore interesting to observe that the silages treated with AD-I and AD-II have reduced levels of aflatoxin B1 and zearalonone, which is a highly desirable property for future applications. Specifically, these two mycotoxins were negatively correlated to rumen motility and infertility, while their acceptable levels in livestock feeds were below 20 μg/kg and 0.56 mg/kg, respectively^20^.

The microbiota profile is another indicator that reflects silage quality. Particularly, good quality silage should not contain any pathogenic bacteria. Consistent with previous studies^21^, the additives used here significantly reduced the populations of pathogenic bacteria that were likely present in the silage raw materials. Some major bacterial species, including *B. megaterium*, *B. marisflavi* and *S. enteric*, may have cause diseases^22^,^23^. The minor bacterial species, *E. durans*, is considered undesirable, even though no report has yet indicated any pathogenic effect of this bacterium on dairy cows. Previous studies have however shown its correlation with antibiotic gene transfer^24^. One possible mechanism of LAB additives in shaping the silage microbiota during the fermentation process was their ability in releasing antagonistic materials^25^. The silage predominant species, *P. acidilactici* and *L. plantarum*, are both capable of producing antibacterial substances that can inhibit the growth of *B. marisflavi*, *S. enteric* and *E. durans*^26^,^27^. Especially, *L. plantarum* was also reported to be able to produce a broad spectrum of antifungal compounds^28^, which could explain the decreases in the silage mycotoxin concentrations after fermentation.

To conclude, the four additive LAB inoculants mostly have positive effects on the fermentation of *M. sativa* silage. Apart from regulating the organic acid and toxin contents, the LAB additives also modulated the bacterial compositions in the fermented silages. Although only eight of the samples were analyzed with the SMRT sequencing technology, our data have shown that this is a prospective method for silage quality assessment.

## Methods

### Silage production

In this study, *M. sativa* planted in the Wuhe city of Anhui province was used. *M. sativa* which was in the budding stage was harvested with a precision chop harvester equipped with an applicator for liquid additives. To reduce the moisture content of the *M. sativa*, the grass was tedded in the sun. As the water content of the *M. sativa* reached 45–50%, the grass was chopped to 1–2 cm. During the cutting process, each of the four different commercial LAB-containing additives (Table 4) were sprayed separately and evenly to the respective chopped *M. sativa* at the concentration of 10^5^ cfu/g. In particular, the first two additives consisted of the same strains, namely *L. plantarum* Ps-8 and *L. plantarum* Ps-9, that were obtained from the Key Laboratory of Dairy Biotechnology and Engineering, Ministry of Education, Inner Mongolia Agricultural University, China. The treated *M. sativa* were then quickly transported with a special truck to the packing site. After unloading from the truck, *M. sativa* was baled and compacted by a strapping machine. Each group consisted of 5 × 600 kg of silage. The fermentation process lasted for 35 days. Samples from the silage, before and after the fermentation, were collected with sterilized containers and were kept in ice boxes during transportation. For the sequencing analysis, protectant was added into the samples to avoid DNA degradation.

### Organic acid analysis and pH measurement

#### Sample preparation

About 225 g deionized water was added into 25 g of silage sample, followed by mixing at 150 rpm for 30 min in a homogenizer. The mixture was acidified with 1 mol/L hydrochloric acid (1:3, w/w) in a centrifuge tube. Then, the homogenate was centrifuged at 9000 rpm for 10 min. Ten milliliter supernatant was filtered through a 0.22 μm pore size membrane filter before chromatographic analysis. For γ-aminobutyric acid analysis, the homogenate was centrifuged at 1 × 10^4^ rpm/min for 5 min.

#### Determination of 4-hyroxy benzoic acid and phenyl lactic acid

Separations with high-performance liquid chromatography (HPLC) were performed on an Agilent 1100 Series LC system. A preparative BEHC18 column (1.7 μm, 2.1 × 100 mm, Waters, America) was used. Solvent A was formic acid diluted in deionized water (1:999), and solvent B was formic acid diluted in acetonitrile (1:999) solution. Elution was performed with a linear gradient as follows: solvent B 20–50% in 2 min, 50–95% in 2.1–3 min, 95–5% in 3–3.1 min. Analytical column temperature was 30 °C, and the flow rate was 0.4 mL/min. The operation conditions for the MS analysis of aflatoxin B1, vomitoxin and zearalonone in positive ionization mode (ESI+) were as follows: capillary voltage, 2.5 kV; cone voltage, 40 V; desolvation gas, 0 L/hr; cone gas, 600 L/hr; source temperature, 100 °C; desolvation temperature, 600 °C.

#### Determination of butyric, acetic and lactic acids

A preparative ZORBAX Elipse AAA C18 column (3.5 μm, 4.6 × 150 mm) was used. Solvent A was phosphate buffer solution (pH 2.5), and solvent B was methanol solution. Elution was performed with a gradient of 97:3. Analytical column temperature was 300 °C, and the flow rate was 1 mL/min. Absorbance was detected at 210 nm.

#### Determination of γ-aminobutyric acid

The o-phthalaldehyde (OPA) derivative reagent was prepared as described previously^29^. Briefly, 10 mg OPA (99%, Sigma) was dissolved in 0.5 mL methanol, then 30 μL 2- mercaptoethanol and 2 mL 0.4 mol/L borate buffer (HPLC grade) (pH 9.4) were added. Before injecting into the machine, 10 μL of sample solution was mixed with 90 μL OPA derivative reagent, reacting for 1 min. A preparative ZORBAX Elipse AAA C18 column (3.5 μm, 4.6 × 150 mm) was used. Solvent A was sodium hydrogen phosphate buffer solution (pH 7.8), and solvent B is the mixture of methanol, acetonitrile and deionized water (45:45:10). Elution was performed with a gradient of 97:3. Analytical column temperature was 35 °C, and the flow rate was 2.0 mL/min. Fluorescence detector was employed for detection with the excitation and emission wavelengths of 340 nm and 450 nm, respectively.

#### pH measurement

25g silage sample was dissolved in 225 mL of deionized water. After vortex mixing for 30 min, a pH meter was used for the measurement.

### Mycotoxin analysis

#### Sample preparation

About 250 g sample was dried to constant weight at 60 °C. After crushing by high speed rotating mill, 25 g crushed sample was transferred to a beaker, followed by adding the mixture of acetonitrile : water (4:6, v/v) in the ratio of 1:8 and leaching for 12 h at room temperature. The mixture was homogenized for 20–30 min in a high speed homogenizer for mycotoxin extraction. Then, it was filtered through a qualitative filter paper. The filtrate was further centrifuged. The resultant supernatant was mixed with acetonitrile and deionized water. After 24 h, the mixture was centrifuged again and the supernatant was collected, enriched by an immunoaffinity chromatography column (BIOTEZ, America) before being analysed with a ultraperformance liquid chromatography–electrospray ionization–quadrupole time-of-flight mass spectrometry (UPLC-ESI-QTOFMS) system (Waters, Milford, MA).

#### UPLC conditions for determination of aflatoxin B1

A preparative ZORBAX Elipse AAA C18 column (3.5 μm, 4.6 × 150 mm) was used. The mobile phase was the mixture of methanol, acetonitrile and deionized water (5:1:1). Analytical column temperature was 30 °C, and the flow rate was 2.0 mL/min. Fluorescence detector was employed with the excitation and emission wavelengths at 235 nm and 460 nm, respectively.

#### UPLC conditions for determination of vomitoxin

A preparative ZORBAX Elipse AAA C18 column (3.5 μm, 4.6 × 150 mm) was used. Solvent A was deionized water, and solvent B was the mixture of methanol and acetonitrile (36:64). Elution was performed with a gradient of 97:3. Analytical column temperature was 35 °C, and the flow rate was 2.0 mL/min. Fluorescence detector was employed with the excitation and emission wavelengths at 235 nm and 460 nm, respectively.

#### UPLC conditions for determination of zearalonone

A preparative C18 column (5 μm, 4.6 × 100 mm) was used. Solvent A was the mixture of methanol, acetonitrile and deionized water (96:2:2), and solvent B was the acetonitrile. Analytical column temperature was 35 °C, and the flow rate was 0.5 mL/min. Absorbance was detected at 220 nm.

#### QTOFMS conditions for determination of mycotoxins

The operation conditions for the MS analysis of aflatoxin B1, vomitoxin and zearalonone in electrospray ionization mode (ESI+) were as follows: capillary voltage, 2.5 kV; cone voltage, 2.5 kV; desolvation gas, 0 L/hr; cone gas, 0 L/hr; source temperature, 100 °C; desolvation temperature, 100 °C.

### SMRT analysis of microbial composition

A total of eight samples, including four each before and after fermentation, were collected, respectively. Sample and sequence information are tabulated (Table 4). DNA was extracted using the OMEGA DNA isolation kit (Omega, D5625-01, USA) following the manufacturer’s instructions. The quality of extracted DNA was checked by 1% agarose gel electrophoresis and spectrophotometry (optical density at 260 nm/280 nm ratio). All extracted DNA samples were stored at −20 °C for further analysis.

The bacterial 16S rRNA was amplified by PCR for barcoded SMRT sequencing with the forward 27F (5′-GAGAGTTTGATCCTGGCTCAG-3′) and the reverse 1541R (5′- AAGGAGGTGATCCAGCCGCA-3′) primers. These primers contained a set of 16-nucleotide barcodes. PCR amplifications of the 16S rRNA regions were performed as described previously^30^. The PCR program was as follows: 95 °C for 2 min; 30 cycles of 95 °C for 30 s, 55 °C for 30 s, and 72 °C for 30 s with a final extension of 72 °C for 5 min. The amplicons were sequenced using P6-C4 chemistry on a PacBio RS II instrument (Pacific Biosciences). The quality control for PCR amplifications and sequence preprocessing was performed as described previously^31^.

Raw data processing was carried out using the protocol RS_ReadsOfinsert.1 available in SMRT Portal version 2.7 as described previously^12^. The extraction of high-quality sequences was firstly performed with the Quantitative Insights Into Microbial Ecology (QIIME) package (version 1.7). Then, PyNAST^32^ and UCLUST^33^ were applied to align the extracted high-quality sequences under 100% clustering of sequence identity to obtain representative sequences. The unique sequence set was classified into operational taxonomic units (OTUs) under the threshold of 98.6% identity using UCLUST after the selection of the representative sequences^34^. The taxonomy of each OTU representative sequence was assigned using the Ribosomal Database Project (RDP) II database that classified at a minimum bootstrap threshold of 80%^35^. A *de novo* taxonomic tree was constructed employing a representative OTU set in FastTree for downstream analysis^36^, including the beta diversity calculation. The Shannon-Wiener, Simpson’s diversity, Chao1 and rarefaction estimators were calculated to evaluate the alpha diversity. The UniFrac distance was calculated based on the phylogenetic tree^34^. Both weighted and unweighted calculations were performed for the principal coordinate analysis (PCoA). The graph presentations were generated by the R package version 3.1.2 and the Origin software version 8.5. The sequence data reported in this study have been deposited in the MG-RAST database (accession No. 4678995.3–4679002.3).

### Statistical analysis

Experimental data were analyzed by the SAS software (SAS version 9.0, SAS Institute Inc. Cary, NC), and the statistical significance was tested by ANOVA. The chemical composition of each sample was tested three times, and the results were expressed as mean ± standard deviation.

## Additional Information

**How to cite this article**: Bao, W. *et al*. Assessing quality of *Medicago sativa* silage by monitoring bacterial composition with single molecule, real-time sequencing technology and various physiological parameters. *Sci. Rep*. **6**, 28358; doi: 10.1038/srep28358 (2016).

## Figures and Tables

**Table 1:** Concentrations of silage organic acids after fermentation.

Item	Samples			
	A^1^	B^1^	C^1^	D^1^
Lactic acid (mg g^−1^)	38.20 ± 0.85^a^	39.10 ± 0.85^a^	39.90 ± 0.90^a^	40.20 ± 1.20^a^
Acetic acid (mg g^−1^)	5.80 ± 0.80^bc^	8.00 ± 1.80^b^	2.10 ± 0.75^c^	13.50 ± 1.92^a^
Acetic acid/lactic acid	0.15 ± 0.02^b^	0.20 ± 0.04^b^	0.05 ± 0.02^c^	0.34 ± 0.04^a^
Butyric acid (mg g^−1^)	0.00 ± 0.00^b^	1.00 ± 0.08^b^	0.00 ± 0.00^b^	5.00 ± 1.01^a^
4-hyroxy benzoic acid (μg g^−1^)	0.20 ± 0.02^b^	0.35 ± 0.05^a^	0.03 ± 0.00^c^	0.06 ± 0.01^c^
Phenyl lactic acid (μg g^−1^)	40.1 ± 1.02^a^	43.34 ± 2.24^a^	35.03 ± 1.13^b^	18.06 ± 0.42^c^
γ-aminobutyric acid (μg g^−1^)	253.12 ± 10.10^a^	214.62 ± 9.53^b^	169.46 ± 8.80^c^	146.19 ± 8.55^c^

^1^A–D: Silage samples treated with four different LAB additives (AD-I, AD-II, AD-III and AD-IV). Within columns, values with different superscript letters are significantly different (*P* < 0.01).

**Table 2:** Mycotoxin concentrations of silage samples before and after fermentation.

Stage	Samples^1^	Mycotoxin (μg g^−1^)^2^		
		Aflatoxin B1	Vomitoxin	Zearalenone
Pre-fermentation	A	0.043 ± 0.002^a^	0.024 ± 0.002^a^	0.103 ± 0.010^a^
	B	0.120 ± 0.011^a^	0.045 ± 0.004^a^	0.063 ± 0.003^a^
	C	0.040 ± 0.003^b^	0.025 ± 0.002^a^	0.115 ± 0.011^a^
	D	0.069 ± 0.006^a^	0.029 ± 0.002^a^	0.045 ± 0.005^b^
Post-fermentation	A	0.039 ± 0.002^a^	0.024 ± 0.003^a^	0.054 ± 0.004^b^
	B	0.107 ± 0.008^a^	0.029 ± 0.002^b^	0.046 ± 0.005^b^
	C	0.080 ± 0.009^a^	0.018 ± 0.001^b^	0.097 ± 0.009^a^
	D	0.084 ± 0.009^a^	0.011 ± 0.002^b^	0.087 ± 0.003^a^

^1^A–D: Silage samples treated with four different LAB additives (AD-I, AD-II, AD-III and AD-IV).

^2^Significant differences in the toxin concentrations between the pre and post-fermentation samples are indicated with different superscript letters (*P* < 0.01).

**Table 3:** Information of sequence and bacterial diversity.

Sample^1^	Number of reads	Number of OTUs	Shannon index	Simpson index	Chao1 index	Observed species
1	4863	3193	10.86	1.00	20516.69	3177.51
2	6847	3360	10.28	1.00	20088.93	3356.32
3	7214	4988	11.76	1.00	32274.75	4941.46
4	7842	3579	9.53	0.98	22334.43	3575.83
5	10760	3432	8.89	0.98	18681.81	3422.74
6	13474	3695	8.46	0.98	21053.80	3684.81
7	7860	2161	7.83	0.97	14693.22	2153.42
8	8515	1628	6.92	0.96	8920.13	1614.82

^1^The first four and the last four numbers represent the samples with LAB additives (AD-I, AD-II, AD-III and AD-IV) before and after fermentation respectively.

**Table 4:** The microbial composition of the LAB additives.

Name of additives	Microbiological composition	Physical form
AD-I	*Lactobacillus plantarum*	Power
AD-II	*Lactobacillus plantarum*	Liquid
AD-III	*Lactobacillus plantarum*, *Lactobacillus casei*, *Enterococcus faecium*, *Pediococcus acidilactici*	Power
AD-IV	*Pediococcus acidilactici*, *Lactobacillus plantarum*	Power
